# When the farm turns fatal: a rare infection causing heart block

**DOI:** 10.1093/omcr/omaf010

**Published:** 2025-03-28

**Authors:** Shaun Abid, Anton Stolear, Maxim Dulgher, Samdish Sethi, Stuart Zarich

**Affiliations:** Department of Internal Medicine, Yale New Haven Health - Bridgeport Hospital, 267 Grant Street, Bridgeport, CT, 06610, United States; Department of Cardiology, Yale New Haven Health - Bridgeport Hospital, 267 Grant Street, Bridgeport, CT, 06610, United States; Department of Internal Medicine, Norwalk Hospital, 34 Maple St, Norwalk, CT, 06850, United States; Department of Cardiology, Yale New Haven Health - Bridgeport Hospital, 267 Grant Street, Bridgeport, CT, 06610, United States; Department of Cardiology, Yale New Haven Health - Bridgeport Hospital, 267 Grant Street, Bridgeport, CT, 06610, United States

**Keywords:** cardiology and cardiovascular systems, infectious diseases and tropical medicine, brucellosis, zoonotic infection, heart block, conduction abnormalities

## Abstract

Brucellosis, a zoonotic infection typically presenting with nonspecific symptoms, rarely leads to cardiac complications, particularly conduction abnormalities. We report a unique case of an 84-year-old female who presented with dizziness, bradycardia, and hypotension, ultimately diagnosed with complete heart block. On admission, blood cultures and further diagnostic workup identified *Brucella* bacteremia as the underlying cause, with further history revealing that she had recently traveled from a farm in Colombia, a brucellosis-endemic area. The patient underwent dual-chamber pacemaker implantation to stabilize her heart rhythm and was initiated on a three-month antibiotic regimen of doxycycline and rifampin. This case highlights the importance of considering brucellosis as a differential diagnosis for heart block in patients with recent travel from endemic regions. Early recognition and intervention, including antibiotic therapy and pacemaker placement when necessary, are essential for achieving favorable outcomes in brucellosis-related cardiac complications.

## Introduction

Brucellosis, a bacterial zoonotic infection caused by *Brucella* species, is primarily transmitted through contact with infected livestock or the consumption of unpasteurized dairy products. It is most common in endemic areas such as the Mediterranean Basin, the Middle East, and Latin America, including Colombia, where our patient had recently traveled [1]. Brucellosis typically presents with nonspecific systemic symptoms, including fever, malaise, and arthralgia, but can occasionally involve multiple organs due to the bacterium’s intracellular persistence and immune-mediated damage [1]. While brucellosis is known for its systemic manifestations, cardiac complications, though rare, have been documented and can be severe. Cardiac involvement includes endocarditis, myocarditis, and, rarely, conduction abnormalities such as complete heart block, with endocarditis being the most common cardiac manifestation and the leading cause of death among brucellosis patients [2]. Given the prevalence of brucellosis in endemic areas, it is crucial to consider this infection in the differential diagnosis of unexplained heart block, particularly in patients with relevant exposure history [3]. Here, we report a case of complete heart block induced by brucellosis in an elderly patient with recent exposure to endemic regions, underscoring the importance of early recognition and management.

## Case presentation

**Figure 1:**
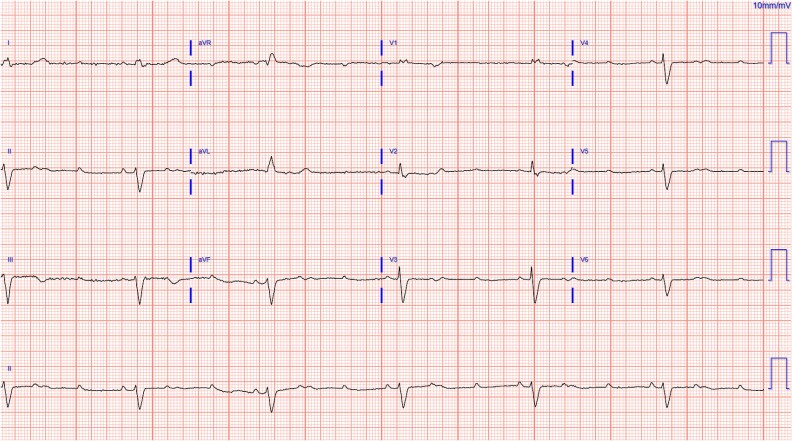
Pre-pacemaker electrocardiogram showing AV dissociation with complete heart block, an atrial rate of approximately 100 beats per minute and a ventricular rate of 35 beats per minute.

An 84-year-old female with a history of diabetes, hypothyroidism, hypertension, and hyperlipidemia presented to the emergency department with complaints of dizziness, episodes of lightheadedness, and blurred vision. On initial assessment, her blood pressure was 86/59 mmHg, and her heart rate was bradycardic at 38 beats per minute. She also experienced orthostatic hypotension and reported exertional shortness of breath without accompanying chest pain, palpitations, or syncope. An initial EKG revealed complete heart block, showing an atrial rate around 100 and a ventricular rate of 35, with intermittent 2:1 conduction (Fig. 1). Review of prior EKGs confirmed a previously normal baseline, highlighting the new onset of complete heart block. Laboratory tests showed normocytic anemia and mild renal impairment, along with an elevated lactic acid level.

Further diagnostic workup included blood cultures, which later returned positive for *Brucella* species, confirming a diagnosis of brucella bacteremia. After this unexpected finding, additional history was obtained from the patient’s family, who reported that she had recently traveled to Colombia, where she spent time on a rural farm. This new information, along with the confirmed brucella infection, suggested zoonotic exposure as the likely source of her infection.

**Figure 2:**
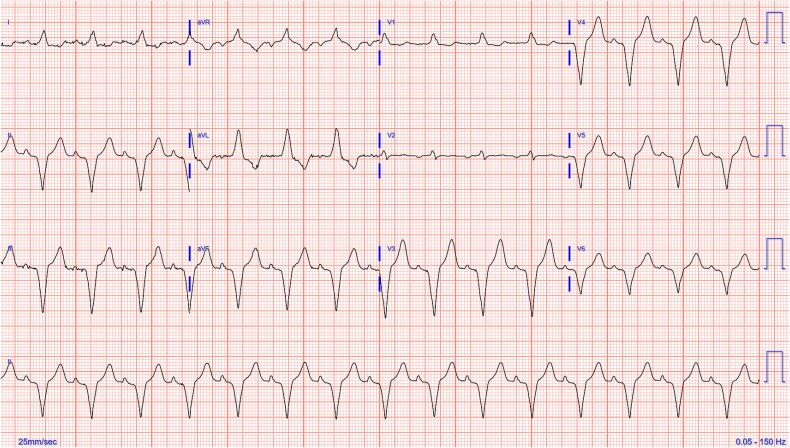
Post dual-chamber pacemaker implantation electrocardiogram demonstrating appropriate A-sensing V-paced pacemaker activity.

Due to symptomatic bradycardia and recurring episodes of severe dizziness, the patient required urgent dual-chamber pacemaker implantation to stabilize her cardiac rhythm (Fig. 2). She was also started on a three-month antibiotic regimen of doxycycline and rifampin to treat the underlying brucellosis. Post-procedure, her bradycardia resolved, with improvements in blood pressure and a reduction in symptoms of lightheadedness. She was discharged with a follow-up plan to monitor her pacemaker function, complete her antibiotic course, and engage in physical therapy to support her recovery from the infection and associated complications. Outpatient management also included regular monitoring of her blood pressure and diabetes to mitigate future cardiovascular risks.

## Discussion

Brucellosis is the most prevalent bacterial zoonosis worldwide, caused by *Brucella* species, which are small, Gram-negative, aerobic, non-motile, non-spore-forming, and unencapsulated coccobacilli [4, 5]. The *Brucella* genus comprises six major species, each associated with a specific principal host: *Brucella melitensis* (sheep and goats), *Brucella abortus* (cattle), *B. suis* (swine), *Brucella ovis* (sheep), *B. canis* (dogs), and *Brucella neotomae* (wood desert rats) [6]. *B. melitensis* is considered the most virulent species for humans and is responsible, along with *B. abortus*, for approximately 70%–75% of brucellosis cases reported annually in the United States, often linked to the consumption of unpasteurized dairy products from endemic regions [6, 7]. A recent study estimated the annual global incidence of human brucellosis to be between 1.6 and 2.1 million cases, highlighting its ongoing significant public health impact [8]. Due to its zoonotic nature and potential for misuse as a biological agent, *Brucella* is classified as a Class B bioterrorism agent [9].

The pathophysiology of cardiac involvement in brucellosis may involve direct bacterial invasion as well as immune-mediated damage, given *Brucella*’s ability to persist intracellularly and invade multiple organs. This can lead to inflammatory responses that disrupt cardiac conduction [10]. Recent studies have shown that brucellosis can lead to alterations in ECG parameters, such as QT dispersion and P-wave dispersion, as well as autonomic imbalance, which may contribute to the risk of arrhythmias and conduction disturbances [11]. In this case, the patient’s complete heart block aligns with these findings, suggesting that brucellosis-induced autonomic dysregulation may have contributed to her condition.

Diagnosis of brucellosis requires a combination of clinical evaluation and laboratory investigations, including blood cultures, serological assays, and molecular methods. However, accurate diagnosis is often challenging due to the nonspecific nature of brucellosis symptoms, which can mimic a range of other infectious and non-infectious conditions, as well as the inherent difficulty in obtaining suitable clinical specimens for testing [12]. Blood cultures, while useful, are complicated by the slow in vitro growth of *Brucella* species, necessitating prolonged incubation of at least four weeks before cultures can be definitively deemed negative [5].

The treatment of brucellosis involves a combination of antibiotics administered for an adequate duration to prevent relapse and ensure complete recovery. For uncomplicated cases in adults and children eight years or older, the recommended regimen includes doxycycline (100 mg twice daily for six weeks) combined with either streptomycin (1 g daily for two to three weeks) or rifampicin (600–900 mg daily for six weeks). In some cases, gentamicin can be used instead of streptomycin, although its effectiveness compared to streptomycin remains uncertain [6]. In this case, the patient experienced complete heart block, necessitating a pacemaker, and was also started on a prolonged (three-month) antibiotic regimen of doxycycline and rifampin to treat the underlying brucellosis.

This case demonstrates a rare instance of complete heart block as a complication of brucellosis, a condition that often presents with nonspecific symptoms but can have significant cardiac involvement in certain cases. Cardiac complications of brucellosis are infrequent, affecting an estimated 1.5% of patients, with endocarditis being the most common manifestation and the principal cause of mortality among brucellosis complications [2]. Although endocarditis is more commonly associated with brucellosis, conduction abnormalities such as complete heart block can also occur and require prompt diagnosis and treatment to prevent life-threatening outcomes. Previous case reports have documented myocarditis and other forms of *Brucella*-induced cardiac damage, which further supports the plausibility of *Brucella* bacteremia leading to severe conduction abnormalities [3, 13]. A recent cross-sectional observational study documented brucellosis as a cause of cardiac conduction disturbances, where early antibiotic intervention was critical in preventing further deterioration of cardiac function, underscoring the importance of timely diagnosis and targeted management in similar cases [11].

This case emphasizes the need for thorough infectious disease screening in patients presenting with unexplained heart block, particularly those with travel history to endemic regions or potential zoonotic exposure. Early antibiotic treatment is critical to managing the infection and preventing complications. In cases where brucellosis leads to symptomatic heart block, pacemaker implantation is often necessary to stabilize cardiac rhythm and prevent further hemodynamic compromise, as seen in this patient. This dual approach—targeted antibiotic therapy and appropriate cardiac intervention—is essential for optimizing outcomes and reducing the likelihood of recurrence or long-term sequelae.

This case highlights the potential for brucellosis to cause complete heart block in patients with recent zoonotic exposure, illustrating the need for early recognition and intervention. Effective management, including antibiotic therapy and timely pacemaker placement, can be critical in preventing adverse outcomes and improving prognosis in cases of brucellosis-related cardiac complications. Given the increasing recognition of brucellosis as a potential cause of conduction abnormalities, this case reinforces the importance of including brucellosis in the differential diagnosis for patients presenting with unexplained heart block, particularly those from endemic areas.
